# 

**Published:** 1879-04

**Authors:** 


### To the Dental Profession.

Six years ago the Maryland Dental College was organized in the city of Baltimore, to meet what was believed to be a serious and pressing want in the line of Dental Education. During its existence of six years it has supplied that want, and steadily fulfilled the object for which it was established. It points with pride to its Alumni, now occupying high positions in the Profession in this and other countries, and to the improvement in the standard of Dental Education wherever its influence has extended.

But there has been for more than a year past a general feeling, which has increased and deepened with passing time and current events, that if the two Dental Colleges located in Baltimore could combine and unite their efforts in the common cause in which both are engaged, their labors would be attended with far greater good to the Profession and larger benefits to all.

This feeling has given rise to negotiations, and these negotiations have resulted in the combination and mergement of the two Institutions, and the uniting of their Faculties. This combination takes the name of the older Institution, "The Baltimore College of Dental Surgery," and the younger Institution carries with it into the combination the peculiar features of its regime, modified and improved by six years of experience.

The Faculty of the Baltimore College of Dental Surgery will issue its diploma to any graduate of the Maryland Dental College, in good standing, who may make application therefor, on the payment of the mere cost of the diploma.

The terms and conditions of the consolidation of the two Colleges have thus been so framed as to protect the rights and interests of all concerned, both now and in time to come; and,—a condition of harmony and concert of action being substituted for one of rivalry,—the friends of both Institutions can unite, as they are hereby cordially invited to do, with each other and with the members of the newly constituted Faculty, and give their encouragement, aid and assistance in elevating the professional standard, by such steps and measures as will secure to every graduate hereafter a thorough and complete Dental Education, which necessarily implies a substantial Medical one. Signed by the following Professors, Regents, &c., of the late

*MARYLAND DENTAL COLLEGE:*

S. H. WILLIAMS, D. D. S., Emeritus Professor.
R. B. DONALDSON, D. D. S., Prof. Dental Surgery.
E. P. KEECH, M. D., D. D. S., Prof. Pathology and Therapeutics.
M. W. FOSTER, M. D., D. D. S., Prof. Dental Mechanism & Metallurgy.
R. B. WINDER, M. D., D. D. S., Prof. of Physiology.
R. FINLEY HUNT, D. D. S., Prof. of Physics and Chemistry.
B. W. BARTON, M. D., Prof. of Anatomy.

| | | |
|---|---|---|
| C. S. HURLBUT, D. D. S., | Regent, | Springfield, Mass. |
| H. H. KEECH, M. D., D. D. S., | " | Baltimore, Md. |
| JAMES F. THOMPSON, M. D., D. D. S., | Regent, | Fredericksburg, Va. |
| H. B. NOBLE, D. D. S., | " | Washington, D. C. |
| GEO. S. FOUKE, D. D. S., | " | Westminster, Md. |
| JESSE C. GREEN, D. D. S., | " | Westchester, Pa. |
| JOHN ALLEN, M. D., D. D. S., | Clinical Instructor, | New York City. |
| W. H. ATKINSON, M. D., D. D. S., | " " | " " " |
| E. PARMLY BROWN, D. D. S., | " " | " " " |
| T. SOLLERS WATERS, D. D. S., | " " | Baltimore, Md. |

### To Readers and Correspondents.

Communications intended for publication, and exchange journals and papers, should be addressed to the EDITOR OF THE AMERICAN JOURNAL OF DENTAL SCIENCE, 259 N. Eutaw St., Baltimore, Md. All letters relating to the business of the journal, or containing cash remittances, to be directed to Messrs. SNOWDEN & COWMAN. Articles for publication should be received by the editor at least three weeks previous to the first day of the month on which the number in which it is designed for them to appear, is issued.

CONTENTS:

ARTICLE I.—Foul Breath. By J. T. Codman, D. D. S. .......... 529
ARTICLE II.—The Use of Chloroform in Dental Operations .......... 542
ARTICLE III.—Valedictory Address. By Rev. Dr. Alex. W. Weddell .......... 552
ARTICLE IV.—Class Address. By Frank B. Perry, D. D. S. .......... 561
ARTICLE V.—Compound Hare-Lip .......... 567

EDITORIAL, ETC.

Close of Volume, Etc .......... 569

OBITUARY.

Prof. John Hugh McQuillen, M. D., D. D. S. .......... 571
Prof. Philip H. Austen, M. D., D. D. S. .......... 576

☞ The American Journal of Dental Science will contain fifty pages of reading matter in each number, making a volume of about

*Six Hundred* pages

EXCLUSIVE OF ADVERTISEMENTS

THE

### AMERICAN JOURNAL

OF

### DENTAL SCIENCE.

The *Journal* is issued on the first of each month and contains not less than *fifty* pages of reading matter in each number, exclusive of advertisements, making a volume of *six hundred* pages per annum.

In each number will be found Original Communications, Correspondence, Selected Articles, Monthly Summary, Bibliographical Notices and Editorial Matter, and every effort will be exerted to render the *Journal* worthy of the patronage of the Dental profession.

A generous co-operation is respectfully solicited from the members of the profession; and as the *Journal* has now a printing office of its own, which materially lessens the cost of its publication, it will be issued at the reduced subscription price of

**$2.50 Per Annum in Advance.**

Communications are respectfully solicited on all subjects pertaining to the practice of dentistry, as well as reports of cases occurring in dental practice.

### RATES OF ADVERTISING.

| | 1 MO. | 2 MO. | 3 MO. | 6 MO. | 12 MO. |
|---|---|---|---|---|---|
| 1 PAGE. | $15 00 | $22 50 | $30 00 | $50 00 | $100 00 |
| ½ " | 10 00 | 15 00 | 20 00 | 30 00 | 50 00 |
| ¼ " | 7 00 | 11 00 | 15 00 | 20 00 | 30 00 |
| ⅛ " | 5 00 | 8 00 | 11 00 | 15 00 | 20 00 |

All original communications designed for publication, works for review, new instruments for notice, exchanges, &c., should be directed to the editor, 259 N. Eutaw St., Baltimore, Md.

All advertisements and subscriptions should be addressed to

SNOWDEN & COWMAN,

*Publishers of the Am. Journal of Dental Science.*

86 W. FAYETTE ST. BET. CHARLES & LIBERTY STS.,

BALTIMORE, MD.

